# Non-consensual Sex and Association with Incident HIV Infection Among Women: A Cohort Study in Rural Uganda, 1990–2008

**DOI:** 10.1007/s10461-012-0378-8

**Published:** 2012-12-05

**Authors:** Isolde Birdthistle, Billy N. Mayanja, Dermot Maher, Sian Floyd, Janet Seeley, Helen A. Weiss

**Affiliations:** 1 Department of Population Studies, Faculty of Epidemiology and Population Health, London School of Hygiene & Tropical Medicine, 15-17 Tavistock Place, London, WC1H 9SH UK; 2 MRC/UVRI Uganda Research Unit on AIDS, Entebbe, Uganda; 3 Department of Infectious Disease Epidemiology, Faculty of Epidemiology and Population Health, London School of Hygiene & Tropical Medicine, 15-17 Tavistock Place, London, WC1H 9SH UK; 4 School of International Development, University of East Anglia, Norwich, NR4 7TJ UK

**Keywords:** HIV, Women, Africa, Non-consensual sex, Intimate partner violence, Cohort study

## Abstract

Non-consensual sex is associated with HIV infection in Africa, but there is little longitudinal data on this association. We describe reported non-consensual sex among women over two decades in southwest Uganda, including associations with incident HIV infection. Between 1990 and 2008, individuals in a population cohort who recently seroconverted to HIV were enrolled into a clinical cohort, along with randomly selected HIV-negative controls. Participants were invited to the study clinic every 3 months, and females asked about recent experiences of sex against their will. Associations of non-consensual sex with HIV status were analyzed prospectively using conditional logistic regression, adjusting for age and year of interview, allowing for within-woman correlation. 476 women aged 14–81 enrolled and attended 10,475 visits over 19 years. The results show high levels of repeated non-consensual sex, often long after HIV infection. There was more reporting among women living with HIV compared to HIV-negative women (22 vs 9 %; OR = 2.29, 95 %CI 1.03–5.09), with the strongest associations among married participants. HIV programmes should address repeated sexual coercion before and subsequent to HIV infection.

## Introduction

High prevalence of HIV among 15–24 year old women has been a persistent feature of HIV epidemics in sub-Saharan Africa [1–5] and has eluded most HIV prevention efforts [6]. The higher prevalence in young women compared with young men may be due to a mix of biological, behavioural and socio-economic factors, including cultural and financial pressures driving intergenerational and non-consensual sex [7].

High levels of non-consensual sex have been documented in hyperendemic HIV settings. For example, in a national survey of 13–24 year old females in Swaziland, one-third reported some form of sexual violence before the age of 18. More specific questions showed that 5 % had ever been “physically forced to have sexual intercourse” and 9 % had ever been “persuaded or pressured to have sexual intercourse against their will” [8]. In the Ugandan Demographic and Health Survey (DHS) in 2006, 21 % of young women and 7 % of men aged 15–19 years reported having ever been “forced to have sexual intercourse or perform other sexual acts against [their] will.”[9] The prevalence rose to 41 % among 20–24 year-old women, and 49 % among 30–39 year olds. In a population-based survey in Nyeri, Kenya, 21 % of females and 11 % of males under 25 years who had ever had sex, reported having been coerced into sex, either through deception, threats, insistence, physical force, being locked in a room, or rape [10].

Reported *recent* non-consensual sex may be less prone to recall bias than lifetime history [11]. In Swaziland, for example, 3 % of 13–17 year old females reported coerced intercourse in the past year [8]. Some studies have asked whether first sex was coerced, as participants are likely to remember the circumstances around their first sexual experience. The Ugandan DHS showed that 24 % of women aged 15–49 reported that their first sexual intercourse was forced against their will, compared to less than 1 % of men. In other contexts, first sex was described as forced among 37 % of young women in Harare, Zimbabwe, and 46 % in KwaZulu Natal [12, 13].

Studies investigating links between coerced sex and HIV are largely based on cross-sectional retrospective studies; many cite significant associations but cannot establish the temporal sequence between violence and HIV [14–16]. Exceptionally, a cohort study in South Africa documented an association between intimate partner violence and incidence of HIV infection, after 2 years of follow up with young women aged 15–26 years [17].

In this paper, we describe patterns of reported non-consensual sex among women enrolled in an open population-based cohort in rural Uganda, from 1990 to 2008. We initially planned to focus on women aged 14–24 years but expanded the age range to include all women because, with up to 18 years of follow up, many young enrollees were still participants after the age of 24 (and some to the age of 42). We thus describe reported non-consensual sex among all women at enrolment and prospectively. We also examine the association of non-consensual sex with incident HIV infection, and differences in this association by age.

## Methods

### Ethics Statement

The General Population Cohort and the Rural Clinical Cohort studies were approved by the Uganda Virus Research Institute Science and Ethics Committee, and the Uganda National Council for Science and Technology.

### Data Collection

In November 1989, a general population cohort (GPC) of approximately 5,000 adults (aged 13 years and above) in 15 villages in rural southwestern Uganda was established. The following year, one-third of those who tested positive for HIV-1 identified in the initial GPC survey were randomly selected and invited to enrol into a rural clinical cohort (RCC) as ‘prevalent cases’ [18]. Annual HIV-1 serosurveys have since been performed on consenting adults, and all seroconvertors (those with a previous negative test within the past 4 years) have been invited to enrol into the RCC as ‘incident’ cases. The cut-off of 4 years was chosen to ensure that incident cases were enrolled within at least 2 years of seroconversion, since the estimated date of seroconversion is taken to be midway between the last negative and first positive HIV-1 test result. HIV-negative controls for the prevalent and incident cases were randomly selected from the GPC, with individual matching on age and sex, with one control per case. There is no replacement selection if the case or the control failed to enroll, or in the event that an HIV-negative control seroconverted after enrolment in the RCC. In 2002, prevention of vertical HIV transmission was introduced in the study clinic and from 2004, anti-retroviral treatment (ART) was phased into the clinical cohort. Further details of the community and clinical cohorts have been published elsewhere [16, 20].

All RCC participants—HIV-positive and HIV-negative—were invited to visit the study clinic every 3 months. Details of enrolment and follow-up procedures within the RCC have been described previously [18]. During these booked appointments, consenting participants were seen by clinicians who administered a detailed socio-demographic, behavioural and medical questionnaire and conducted a physical examination. At their enrolment visit, all female participants were asked: “In the last 12 months, have you ever had sex against your will?” In all subsequent visits, they were asked: “Since your last visit, have you ever had sex against your will?”

Clinic staff were blinded to participants’ HIV status up to the introduction of ART in January 2004.

### Laboratory Methods

All sera were tested using two enzyme immunoassay (EIA) systems: Recombigen HIV-1 EIA (Cambridge Biotech Corporation, Worcester, MA, USA) and Wellcozyme HIV-1 Recombinant (Wellcome Diagnostics, Dartford, UK). Western blot (Novopath HIV Immunoblot, Bio-Rad laboratories, Watford UK) was used when the EIAs gave discrepant results.

### Data Analysis

Data were entered in FoxPro up to 2004 and MS Access (Microsoft Corp, USA) thereafter, and analysed in *Stata*, version 11 (Stata Corporation, East College Station, TX USA). Analysis was restricted to women who enrolled in the RCC between 1990 and 2008.

We first considered whether non-consensual sex is a risk factor for incident HIV infection, taking a case–control approach. For women with incident HIV infection (n = 188), we used data from the first visit at which they were recognised to be HIV positive, and for HIV negative women (n = 166), we used data from the enrolment visit, and calculated the proportions who reported non-consensual sex during the previous 12 months. Incident HIV cases and matched controls were stratified retrospectively by age-group and year of enrolment. These strata formed the matched sets for conditional logistic regression, to produce odds ratios controlled for age and year of enrolment. Other sexual risk behaviours were assessed among the young women—such as early sexual debut and number of lifetime sexual partners—to help validate the self-reported non-consensual sex, and to understand HIV risk among the young participants in greater detail.

We also considered whether the risk of non-consensual sex is higher among women living with HIV compared to HIV-negative women. Using longitudinal data from all clinic visits (enrolment and follow-up), reports of non-consensual sex were summarised among HIV-negative (n = 4,364 visits) and HIV positive (n = 3,143) women (both incident and prevalent). Logistic regression was used to investigate whether HIV positive women were at increased risk of non-consensual sex compared to HIV-negative women, quantifying the association using odds ratios, controlling for age and calendar period at interview and allowing for within-woman correlation using robust standard errors. Associations of HIV status with non-consensual sex were also assessed by marital status, with women describing their relationship status as either ‘married’, in a ‘steady relationship’, divorced, separated, widowed or single.

## Results

### Characteristics of the Cohort at Enrolment

From 1990 to 2008, 476 women aged 14–81 years were enrolled in the RCC. Overall, 166 (35 %) participants were HIV-negative and 167 (35 %) were confirmed incident HIV cases at the time of enrolment (Table 1). The distribution of newly-enrolled participants in terms of age, gender and HIV status changed over the course of the RCC, partly reflecting changes in the method and ratio of matching. During follow-up, 21 women in the RCC seroconverted to HIV, resulting in a total of 188 HIV incident cases by 2008.

**Table 1:** General characteristics of female participants at the time of enrolment

	All n (%)	HIV-negative controls n (%)	Incident HIV-positive cases n (%)
Total	476	166 (34.9)	167 (35.1)
Age at enrolment (years)			
14–19	54 (11.3)	15 (9.0)	31 (18.6)
20–24	100 (21.0)	40 (24.1)	42 (25.2)
25–29	73 (15.3)	27 (16.3)	24 (14.4)
30–39	119 (25.0)	36 (21.7)	39 (23.4)
40–49	69 (14.5)	20 (12.1)	16 (9.6)
50–81	61 (12.8)	28 (16.9)	15 (9.0)
Year of birth			
1909–1959	130 (27.3)	61 (36.8)	29 (17.4)
1960–1969	137 (28.8)	54 (32.5)	30 (18.0)
1970–1979	150 (31.5)	42 (25.3)	72 (43.1)
1980–1991	59 (12.4)	9 (5.4)	36 (21.6)
Marital status			
Single	44 (9.3)	17 (10.2)	16 (9.6)
Married	232 (48.8)	100 (60.2)	80 (48.2)
Divorced/sep/widowed	159 (33.5)	39 (23.5)	46 (27.7)
Steady partner	40 (8.4)	10 (6.0)	24 (14.5)
Year of enrolment (in relation to changes in selection procedures and the provision of treatment within the study)			
Pre-treatment 1990	73 (15.3)	38 (22.9)	0
1991–1994	103 (21.6)	70 (42.2)	26 (15.6)
1995^a^–2001	80 (16.8)	28 (16.9)	47 (28.1)
PMTCT^b^: 2002–2003	36 (7.6)	7 (4.2)	29 (17.4)
ART^b^: 2004–2008	184 (38.7)	23 (13.9)	65 (38.9)

^a^Selection procedure changed from 1:1 to 1 HIV-negative control enrolled for every 2 new incident cases

^b^PMTCT/ART introduced in the study clinic, offered to HIV-positive participants

Due to the differences in characteristics of the RCC participants over time, subsequent analyses were stratified by enrolment year. Analyses were also stratified by age and marital status due to particular interest in their effect on the relationship between non-consensual sex and HIV. Comparisons were restricted to HIV-negative and incident HIV cases, as the timing of HIV sero-conversion could not be known for the prevalent HIV cases and findings would be difficult to interpret.

### Non-consensual Sex as a Risk Factor for Incident HIV at Enrolment

Table 2 presents associations between recent non-consensual sex and incident HIV infection, using reports from enrolment (non-consensual sex in the past 12 months), or the first seropositive visit for individuals who seroconverted after enrolment in the cohort (non-consensual sex since the last visit). A higher proportion of incident HIV-positive than HIV-negative females reported recent non-consensual sex (24 vs 16 %; unadjusted OR = 1.64, *p* = 0.089). However, this relationship was no longer significant after adjusting for age and enrolment year [adjusted OR (aOR) = 0.93, CI 0.47–1.82]. Among young women aged 14–24 years, there was no evidence that being HIV-incident was associated with having initiated sex before age 16 (aOR = 1.58, CI 0.65–3.82), or with reporting two or more lifetime partners (aOR = 2.10, CI 0.80–5.40), compared with HIV-negative women.

**Table 2:** Associations between sexual experiences and incident HIV among women at enrolment (or time of seroconversion)

	HIV-negative controls n/166 (%)	Incident HIV cases n/188 (%)^a^	Unadjusted OR (95 % CI)	Adjusted OR^b^ (95 % CI)
*Recent coerced sex, all females*				
No	122 (84.1)	133 (76.4)	1	1
Yes	23 (15.9)	41 (23.6)	1.64 (0.93–2.88)	0.93 (0.47–1.82)
If aged 14–19 at enrolment				
No	9 (90.0)	21 (70.0)	1	1
Yes	1 (10.0)	9 (30.0)	3.86 (0.42–35.1)	3.22 (0.36–29.1)
If aged 20–24 at enrolment				
No	27 (73.0)	30 (75.0)	1	1
Yes	10 (27.0)	10 (25.0)	0.9 (0.32–2.49)	0.37 (0.11–1.30)
If aged 25–29 at enrolment				
No	25 (92.6)	17 (73.9)	1	1
Yes	2 (7.4)	6 (26.1)	4.41 (0.79–24.51)	1.70 (0.22–12.90)
If aged 30–39 at enrolment				
No	29 (82.9)	32 (86.5)	1	1
Yes	6 (17.1)	5 (13.5)	0.76 (0.21–2.74)	0.40 (0.10–1.65)
If aged 40–49 at enrolment				
No	16 (84.2)	9 (64.3)	1	1
Yes	3 (15.8)	5 (35.7)	2.96 (0.57–15.39)	3.31 (0.46–23.68)
If aged 50–81 at enrolment				
No	16 (94.1)	8 (80.0)	1	1
Yes	1 (5.9)	2 (20.0)	4.00 (0.31–51.03)	4.00 (0.18–87.32)
*Other sexual experiences among young women (enrolled when 14*–*24* *years old)*				
Early sexual debut				
At 16+ years	31 (60.8)	40 (56.3)	1	1
Before 16 years	20 (39.2)	31 (43.7)	1.20 (0.58–2.50)	1.58 (0.65–3.82)
Lifetime sexual partners				
1	19 (39.6)	19 (27.1)	1	1
2	16 (33.3)	28 (40.0)	1.75 (0.72–4.24)	2.0 (0.70–5.81)
3 or more	13 (27.1)	23 (32.9)	1.77 (0.70–4.49)	2.21 (0.72–6.77)

^a^odds ratio calculated using conditional logistic regression, stratified on age and enrolment year

^b^The 21 females who seroconverted *during* the RCC were classified as both HIV negative Controls (at enrolment) and incident HIV Cases (at the time of seroconversion). Their respective answers to the question on non-consensual sex were taken from their enrolment interview and the interview preceding seroconversion. (Sensitivity analyses showed that exclusion of the sero-converted cases did not change the results)

### Self Reported Experiences of Recent Non-consensual Sex at Each Visit

Among the 476 females enrolled, 445 (94 %) returned for at least one follow-up visit and the median number of visits was 45 (maximum = 73). The maximum length of time participants were followed was 18 years (n = 28 women).

Women were asked about new episodes of non-consensual sex at each follow-up visit. Overall, 27 % of women who visited more than once (119/438 for whom data were available) reported an experience of coerced sex in at least one visit. The number of times participants reported non-consensual sex ranged from 1 to 57. The youngest woman to report recent coerced sex was 16 years old and the oldest was 62.

Among the 119 women who ever reported non-consensual sex, 80 (67 %) reported it more than once, including 57 women (48 %) who reported it in at least half of their visits and 25 (21 %) at all their visits. Women aged 14–19 at enrolment were the most likely to report non-consensual sex in all visits (36 %), followed by those aged over 40 years (30 %). Overall, there was high within-woman correlation of reporting over time, with only 22 % of women changing their reporting of non-consensual sex during follow-up visits.

### Differences in Reporting Non-consensual Sex by HIV Status at Each Visit

**Fig. 1:**
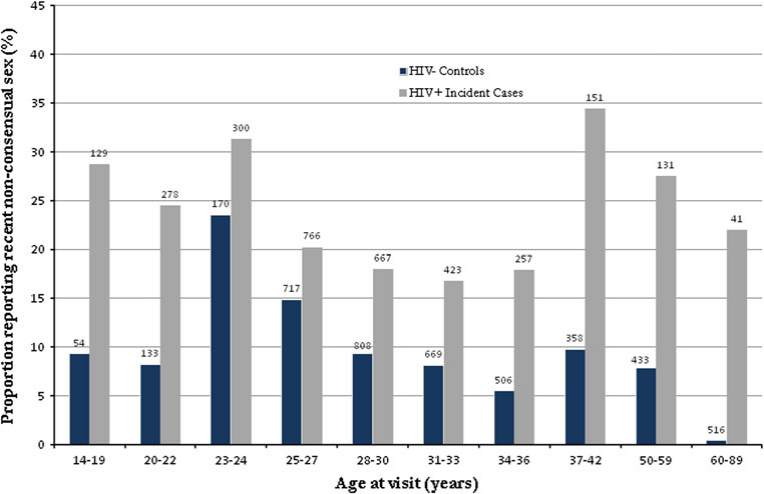
Prevalence of recent non-consensual sex reported by age at visit (N = 7,507 visits). The *number above each* *bar* represents the total number of visits by participants in each age group

During follow up, recent non-consensual sex was reported more frequently by participants who had been enrolled as HIV-incident cases (at 22 % of all visits) than by HIV-negative controls (9 %; aOR = 2.29, 95 %CI 1.03–5.09), and this was true in every age group (Fig. 1).

Among HIV-negative participants, 9 % of the youngest participants (14–22 year olds, grouped due to very small differences between 14–19 and 20–22 year olds) reported non-consensual sex, rising to 24 % among 23–24 year olds. Prevalence declined from age 25, to relatively low levels among the oldest age groups (e.g., 4 % of visits by 50–89 year olds). In contrast, among women who had been enrolled as incident HIV cases, reported non-consensual sex was highest among the youngest (26 % among 14–24 year olds) and oldest ages (45 and older).

When assessed by marital status, associations of incident HIV with non-consensual sex were strongest among married participants, among whom 33 % of HIV cases and 11 % of HIV negative participants reported recent non-consensual sex (aOR = 3.46; 95 %CI 1.45–8.28). Among women in steady relationships, those who had enrolled as incident HIV cases were also more likely than HIV-negative participants to report recent non-consensual sex (16 vs 0 %). Among single women, HIV-positive participants were not more likely than HIV-negative women to report recent non-consensual sex (approximately 11 % of all single women reported recent non-consensual sex).

When assessed by calendar year in Table 3, associations of HIV with non-consensual sex were strongest among participants interviewed in earliest years of the study (1990–1994). While reporting of non-consensual sex among HIV-positive women did not vary considerably over time, it increased among HIV-negative women. Specifically, 3.3 % of HIV-negative women reported non-consensual sex in 1990–1994 compared to 11 % after 2002.

**Table 3:** Associations between HIV status and reporting recent experience of non-consensual sex (N = 7,507 visits)

Reported recent experience of non-consensual sex	HIV-neg’ve participants (N = 4,364 visits) n (%)	Incident HIV cases (N = 3,143 visits) n (%)	aOR (95 % CI)*
All	391 (9.0)	688 (21.9)	2.29 (1.03–5.09)**
Stratified by age at interview, adjusted for year of interview			
Age 14–22 (594 obs)	16/187 (8.6)	105/407 (25.8)	2.28 (0.48–10.78)
Age 23–24 (533 obs)	40/170 (23.5)	94/300 (31.3)	0.99 (0.28–3.45)
Age 25–29 (1,652 obs)	107/717 (14.9)	155/766 (20.2)	1.28 (0.39–4.26)
Age 30–39 (3,202 obs)	129/1,477 (8.7)	191/1,090 (17.5)	1.89 (0.64–5.59)
Age 40–44 (1,028 obs)	28/506 (5.5)	46/257 (17.9)	4.08 (1.06–15.65)
Age 45–49 (635 obs)	35/358 (9.8)	52/151 (34.4)	5.49 (0.83–36.31)
Age 50–89 (1,308 obs)	36/949 (3.8)	45/172 (26.2)	9.51 (0.92–98.87)
Stratified by year of interview, adjusted for age at interview			
1990–1994 (1,380 obs)	27/831 (3.3)	41/186 (22.0)	16.20 (2.90–90.50)
1995–2001 (3,499 obs)	176/1,857 (9.5)	31/1,279 (24.6)	2.49 (0.91–6.83)
2002–2003 (950 obs)	65/531 (12.2)	110/419 (26.3)	1.77 (0.65–4.87)
2004–2008 (3,128 obs)	123/1,145 (10.7)	222/1,259 (17.6)	1.44 (0.72–2.87)
Stratified by marital status at interview, adjusted for age at interview and year of interview			
Single	25/216 (11.6)	24/229 (10.5)	0.43 (0.03–7.30)
Married	286/2,579 (11.1)	429/1,304 (32.9)	3.46 (1.45–8.28)
Divorced/sep^a^/widowed	80/1,318 (6.1)	137/1,008 (13.6)	1.50 (0.20–11.06)
Steady partnership	0/251	98/597 (16.4)	–

* Adjusted as specified and allowing for within-woman correlation

** Adjusted for age at interview and year of interview, allowing for within-woman correlation

^a^Separated

The strength of associations weakened when allowing for within-woman correlation (changes not shown). Correlation in reporting was explored to understand its role in explaining differences in experiences of non-consensual sex by HIV status. As shown in Table 4, of the women who ever reported coerced sex, a minority did so only once (26 % of HIV-negative and 17 % of incident HIV cases), and substantial proportions in both groups reported coerced sex at least 10 times (31 and 46 % respectively; *p* = 0.16 for linear trend).

**Table 4:** Associations between repeated reporting of non-consensual sex and HIV status (N = 83)

Times reported non-consensual sex*	HIV-negative controls (n = 35) n (%)	Incident HIV cases (n = 48) n (%)	Test for linear trend
Once only	9 (25.7)	8 (16.7)	*p* = 0.16
2–9 times	15 (42.9)	18 (37.5)	
10 or more times	11 (31.4)	22 (45.8)	

* Among women who ever reported non-consensual sex and visited more than once

## Discussion

This study is one of few to capture experiences of non-consensual sex and HIV infection prospectively rather than retrospectively. In addition, the long follow-up period and frequent visits enabled us to track trends in reporting non-consensual sex by age and calendar time, as well as by HIV status. These data offer insight into the links between non-consensual sex and HIV in a number of important ways.

First, recent non-consensual sex was reported by women across a wide age range (16–62 years). There were high levels of repeated sex against one’s will, with many women—particularly women who were married or in steady relationships—reporting new episodes of non-consensual sex in most or all of their visits.

In all years, reports of recent non-consensual sex were higher among women enrolled as incident HIV cases than HIV-negative participants, and particularly among the youngest and oldest HIV positive women. Previous studies of sexual coercion and HIV have not captured the experiences of older women. The only known cohort study on sexual violence and HIV was limited to women under 25 years [17], and the DHS [9] and WHO Multi-Country Study on Women’s Health and Domestic Violence [19]—which capture experiences of sexual violence in large population-based samples—are limited to women under 49, and do not compare reporting among HIV positive and HIV negative women.

In terms of the temporal sequence between non-consensual sex and HIV, there were high levels of unwanted sex both at the time of and after HIV seroconversion. With substantial follow up after seroconversion, the analysis of all visit data (over 7,000 follow-up interviews) revealed high levels of non-consensual sex among women living with HIV/AIDS (more than twice that of women who remained HIV negative through the study). And across the study period, new episodes of non-consensual sex were often reported long after seroconversion.

We also sought to identify non-consensual sex as a risk factor for HIV infection. Reporting non-consensual sex before or at the time of HIV seroconversion was higher than that among HIV-negative participants; however, the evidence did not reach statistical significance. In an earlier case–control analysis with the first 133 women enrolled in the RCC between 1990 and 1997, incident cases (30 %) were significantly more likely than seronegative controls (4 %) to report non-consensual sex in the 12 months prior to their enrolment interview (OR = 7.84; 1.29–47.86) [16]. The smaller difference between cases and controls in the current and longer-term analysis may be explained by the larger proportion of HIV-negative women (16 %) who, by 2008, had reported recent non-consensual sex prior to enrolment. The analysis by calendar year showed that, consistent with the previous analysis by Quigley and colleagues, the association between non-consensual sex and HIV was strongest in the early years of the study (particularly 1990-1994) and subsequently decreased as reporting of non-consensual sex increased among HIV-negative women. This may reflect a real increase in non-consensual sex and/or changes in reporting over time. If true, the finding that many HIV-negative women report non-consensual sex suggests a changing effect of coerced sex on HIV over time, e.g., a diminished effect when HIV becomes more established in the general population.

We were initially interested in non-consensual sex among young women, as a possible explanation for high HIV prevalence among 15–24 year old females compared to males. We found a high prevalence of reported non-consensual sex in the past 12 months among young HIV-negative participants (23 % among 14–24 year olds). This is consistent with reporting of unwanted sex in other population-based studies, although variability in the measurement of unwanted sex impedes direct comparisons. For example, most studies ask about any lifetime experience of non-consensual sex, which should result in higher prevalence than our study of recent experience. The nationally representative Uganda DHS 2006, for example, found that 30 % of young women aged 15–24 years had ever experienced ‘sexual violence’ (which includes but is not limited to forced sexual intercourse).

By following young women over time, this study showed that experiences of coerced sex—often experienced for the first time in adolescence—can recur frequently and over many years. Jewkes and colleagues [17] found higher HIV incidence with more frequent partner violence, and hypothesise that such ‘chronically abusive’ relationships have more effect on HIV transmission than single events. This study shows that such repeated abuse can continue long after HIV infection.

### Limitations

The question, “have you had sex against your will” tells us whether a woman had sex that was not wanted, but does not tell us why. There is a range of reasons why sex may not be consensual, including physical force, persuasion, bribery, fear or marital obligation. In this study, we do not know what particular circumstances led a participant to answer ‘Yes’, or how harmful her situation. Also, the question of ‘sex against your will’ may be interpreted differently by different women, and, given the length of the study, prompts or guides may not have been consistent across interviewers or time. Furthermore, there are reasons why women may ‘over-report’ coercive sex, e.g., to avoid blame for behaviour viewed as unacceptable or to explain a pregnancy [20], or ‘under-report’ unwanted sex, e.g., for fear of implicating their partner. Social desirability bias may act in different ways at different ages (e.g., if sexual behavior is disapproved among adolescents and/or older, non-reproductive women), or by HIV status (e.g., to avoid blame for being HIV-positive). There may also be systematic reasons why reporting of non-consensual sex differs by HIV status, for example, declining health and libido among women living with HIV/AIDS, and thus less ‘willingness’ to have sex, compared to HIV-negative women. The questionnaire and study design thus limited our investigation of the meaning and context of the women’s responses.

This analysis could not provide evidence of a causal association between non-consensual sex and HIV infection and this may be due to a changing effect of the HIV epidemic over time (as discussed above), or the following design limitations. First, the enrolment interview only captured incidents of non-consensual sex up to 1 year before enrolment in the cohort, and there may have been episodes before that time (the high levels of repeated coerced sex after enrolment suggests this is likely). Further, there was uncertainty around the precise timing of seroconversion, as it was estimated as the mid-point between the last negative and first positive HIV test, which often spanned several years. These limitations are likely to have underestimated the effect of non-consensual sex on HIV infection.

While consistent patterns emerge from analysis of the very large amount of visit data after enrolment, there is weak evidence of statistical associations in specific age/calendar group analysis, given the relatively small number of individuals for sub-group analyses.

Drawing from a general population study should have helped to minimize selection bias, in that there was little likelihood the women’s selection was related to their experiences of non-consensual sex. Focusing on ‘recent’ experiences of non-consensual sex should have helped to minimize recall bias, although there is the possibility that HIV-positive women will remember such experiences, or when such events happened, more than HIV-negative women. Finally, it is possible that observed associations between non-consensual sex and HIV status are partially explained by factors which were not measured in this study (residual confounding).

### Implications

The findings add to evidence that HIV prevention programmes must incorporate issues of sexual coercion and consent, from early adolescence. But efforts should not stop there. Women living with HIV should be protected from repeated sexual coercion, which this study shows can persist long after becoming HIV positive. In many parts of sub-Saharan Africa, where levels of serodiscordance are high and HIV disclosure often low, agencies involved in protecting women from violence should be linked with programmes promoting HIV testing and counseling, HIV disclosure to partners, and partner participation in PMTCT.

In terms of future research, women over 49 years are also under-represented in current HIV and sexual health research, and we urge closer attention to their sexual experiences, risks and well-being. Much could be learned by expanding the age range of large population-based studies, as has been done in other contexts (e.g., the National Survey of Sexual Attitudes and Lifestyles in the UK has recently broadened its upper age limit to 74). Operational and evaluation research can investigate whether women in early adolescence and older women are missed by HIV and sexual services that target women of reproductive age.

Finally, we strongly recommend qualitative research in this area to elucidate the continuum between coercion and consent, e.g., under what circumstances women consider sex to be against their will, and how such experiences, including gender equity in relationships, vary by age and HIV status. Qualitative research can also help illuminate the role of sexual violence in the lives of women living with HIV, including decisions around HIV testing, disclosure and adherence to treatment.
